# Computerized decision support system improves consistency of haemodynamic assessment amongst ICU team members

**DOI:** 10.1186/cc12137

**Published:** 2013-03-19

**Authors:** AE Aneman, R Ranganatha

**Affiliations:** 1 Liverpool Hospital, Liverpool, Australia

## Introduction

This study evaluated the potential of the NAVIGATOR (Applied Physiology) haemodynamic monitor to improve consistency of cardiovascular assessments amongst ICU members with different levels of expertise and experience.

## Methods

Patients (*n = *20) post cardiac surgery were monitored by NAVIGATOR to display heart efficiency (Eh), mean systemic filling pressure (Pms) and vascular resistance (SVR) against targets for mean arterial pressure and cardiac output set by the clinical team. Four categories of staffparticipated: nine consultants (C), eight senior registrars (SR), nine registrars (R) and 11 nurses (N) (median ICU experience 15, 7, 2 and 10 years, respectively) and were asked to score Eh, Pms, and SVR ranging (discrete steps of one) from -5 (grossly subnormal) to 0 (normal) to 5 (grossly supranormal) first without (BLIND) and then given (OPEN) access to the NAVIGATOR display. Recommendations for therapeutic interventions were noted. Agreement (maximum two steps deviation for each assessment between staff; number of patients), disagreement (median and interquartile range of steps) and therapeutic agreement (intervention/s to change Eh, Pms and SVR in similar direction/s, number of patients) were recorded and analysed for statistical difference BLIND versus OPEN (Fisher's exact test, Mann-Whitney test, *P <*0.05).

## Results

Eh was commonly overestimated, Pms commonly underestimated with no clear trend for SVR. Agreement amongst all categories of staff increased and disagreement score decreased for Eh, Pms and SVR (Table 1) comparing BLIND versus OPEN assessments. Agreement for therapeutic interventions also increased significantly from 4/20 (BLIND) to 18/20 patients (OPEN).

**Table 1:** 

	BLIND	OPEN
Eh	6/20, 2 (1 to 3)	17/20*, 1 (0 to 1)*
Pms	6/20, 2.5 (2 to 4)	17/20*, 2 (1 to 2)*
SVR	8/20, 2 (1 to 2)	16/20*, 1 (1 to 2)*

Patients (*n*/20) and score (median and IQR). *P <0.05

## Conclusion

The assessment of heart function, intravascular filling and resistance state in postoperative cardiac patients was made significantly more consistent amongst different ICU staff members using the NAVIGATOR haemodynamic monitor. Such enhanced consistency could potentially make the haemodynamic management more effective with improved clinical outcomes.

